# Oxidized phospholipids are ligands for LRP6

**DOI:** 10.1038/s41413-018-0023-x

**Published:** 2018-07-18

**Authors:** Lei Wang, Yu Chai, Changjun Li, Haiyun Liu, Weiping Su, Xiaonan Liu, Bing Yu, Weiqi Lei, Bin Yu, Janet L. Crane, Xu Cao, Mei Wan

**Affiliations:** 1 Department of Orthopaedics and Traumatology, Nanfang Hospital, Southern Medical University, Guangzhou, Guangdong China; 2 Department of Orthopaedic Surgery, Johns Hopkins University School of Medicine, Baltimore, 21205 MD USA; 3 Department of Endocrinology, Endocrinology Research Center, The Xiangya Hospital of Central South University, Changsha, Hunan China; 4 Department of Dermatology, Johns Hopkins University School of Medicine, Baltimore, MD USA; 5 Department of Pediatric Endocrinology, Johns Hopkins University School of Medicine, Baltimore, MD USA

## Abstract

Low-density lipoprotein receptor–related protein 6 (LRP6) is a co-receptor for Wnt signaling and can be recruited by multiple growth factors/hormones to their receptors facilitating intracellular signaling activation. The ligands that bind directly to LRP6 have not been identified. Here, we report that bioactive oxidized phospholipids (oxPLs) are native ligands of LRP6, but not the closely related LRP5. oxPLs are products of lipid oxidation involving in pathological conditions such as hyperlipidemia, atherosclerosis, and inflammation. We found that cell surface LRP6 in bone marrow mesenchymal stromal cells (MSCs) decreased rapidly in response to increased oxPLs in marrow microenvironment. LRP6 directly bound and mediated the uptake of oxPLs by MSCs. oxPL-LRP6 binding induced LRP6 endocytosis through a clathrin-mediated pathway, decreasing responses of MSCs to osteogenic factors and diminishing osteoblast differentiation ability. Thus, LRP6 functions as a receptor and molecular target of oxPLs for their adverse effect on MSCs, revealing a potential mechanism underlying atherosclerosis-associated bone loss.

## Bone loss: revealing a molecular cause of ‘numb’ stem cells

A constituent of oxidized ‘bad cholesterol’ blocks essential signaling processes leading to pathogenic bone loss. LRP6 is a crucial receptor involved in multiple physiological processes, including bone loss; however, direct pathogenic modulators of the receptor have yet to be elucidated. Now, John Hopkins University School of Medicine’s Mei Wan, with a US and Chinese research team, has discovered that oxPL, a byproduct of the oxidization of cholesterol carrier LDL, binds directly to LRP6 and causes its removal from the surface of bone marrow stem cells. As a result, these stem cells are unable to sense the external signaling molecules that drive bone growth. oxPLs are products of diseases such as hyperlipidemia and atherosclerosis, and this paper helps to reveal their pathogenesis and offers potential targets for therapeutic interventions.

## Introduction

Low-density lipoprotein receptor–related protein 6 (LRP6) is a member of the low-density lipoprotein (LDL) receptor family, which together with LRP5 function as a co-receptor for Wnt/β-catenin signaling.^1–3^ Upon binding of Wnt to its receptor Frizzled, LRP5/6 is recruited to form a complex with Wnt-Frizzled, eventually leading to downstream β-catenin protein stabilization.^4,5^ In addition to its role in mediating Wnt/β-catenin signaling, LRP6 functions as an essential element in signaling pathways activated by multiple hormones/ growth factors. LRP6 has been found to form a complex with parathyroid hormone (PTH) and its receptor PTH1R in osteoblastic lineage cells, facilitating β-catenin-Tcf/Lef signaling^6–9^ and cAMP-protein kinase A (PKA) signaling activation.^7,10–12^ LRP6 also mediates the activation of cAMP-PKA by other G protein–coupled receptor ligands including isoproterenol/β-adrenergic receptor, adenosine/adenosine receptors, and glucagon/glucagon receptor.^10^ Moreover, LRP6 was recognized recently as a co-receptor for platelet-derived growth factor (PDGF)/ PDGF receptor β and transforming growth factor β (TGFβ)/TGF-β receptor 1, negatively regulating their signaling activation in pericytes and myofibroblasts.^13,14^ Thus, LRP6 is a unique co-receptor that mediates the intracellular signal transduction of multiple factors. However, all of these factors (i.e. Wnt, PTH, PDGF, and TGFβ) have their own receptors. Furthermore, direct interaction of these factors with LRP6 in the absence of their own receptors has not been recognized, indicating that they are not actual ligands of LRP6.

Mutations in LRP6 have been linked to early onset hypercholesterolemia, atherosclerosis, and osteoporosis in humans.^15,16^ Additionally, epidemiological studies have shown a positive correlation of osteoporosis to hyperlipidemia and consequent atherosclerosis, independent of age. Specifically, 63% of osteoporotic patients have hyperlipidemia.^17^ People with high LDL levels are also more likely to suffer a non-vertebral fracture.^18^ Aortic calcification, an established marker for atherosclerosis, positively correlates with osteoporosis and fracture risk,^19,20^ and low bone mineral density is used as a clinical marker for atherosclerotic coronary artery disease.^21,22^ As a complex lipoprotein, LDL comprises one molecule of apolipoprotein B-100 and hundreds of molecules of cholesterol and phospholipid,^23^ which are susceptible to enzymatic and free radical oxidation. In the environment of a high levels of reactive oxygen species (ROS), LDL particles undergo varying degrees of oxidation of their phospholipids, resulting in oxLDL, which serves as a hallmark for hyperlipidemia and atherosclerosis.^24^ Carrying oxidized phospholipids (oxPLs), oxLDL induces multiple responses that are detrimental to cells. Within oxLDL, the oxidized forms of 1-palmitoyl-2-arachidonoyl-sn-glycero-3-phosphocholine are the major oxPLs, which contain isolated bioactive components such as POVPC, PGPC, and PEIPC. Whereas native LDL (nLDL) binds to the LDL receptor (LDLR), oxLDL and individual oxPLs bind to several scavenger receptors (SRs), including CD36,^25^ SR-A,^26^ and -B,^27^ the lectin-like oxidized-(LDLR 1 (LOX-1),^28^ and Toll-like receptor 4 (TLR-4)^29^ for the uptake of oxLDL by macrophages and vascular cells to facilitate atherosclerosis development.^30^ It has been recognized that the same oxPLs that promote atherosclerosis also act on bone to exert their detrimental effect.^1,31–33^ However, the major molecular targets of bioactive oxPLs for their adverse effect on bone cells remain elusive. Given that LRP6 is a positive regulator for osteoblastic bone formation,^7,34,35^ and that patients with autosomal dominant LRP6 mutations developed simultaneous early onset atherosclerosis and osteoporosis, it is possible that oxPLs directly target LRP6 in bone cells during hyperlipidemia/atherosclerosis.

In the present study, using animal models and various cell based biochemical approaches, we identify oxLDL particle and individual bioactive oxPLs as native ligands that specifically bind LRP6 on cell surface and induce its endocytosis in bone marrow MSCs, leading to blunted responses of MSCs to osteogenic factors for osteoblastogenesis. The finding offers new understanding on the role of LRP6 in the pathogenesis of atherosclerosis and associated osteoporosis.

## Results

### Cell surface LRP6 is reduced in bone marrow MSCs under microenvironment of high oxPLs

**Fig. 1:**
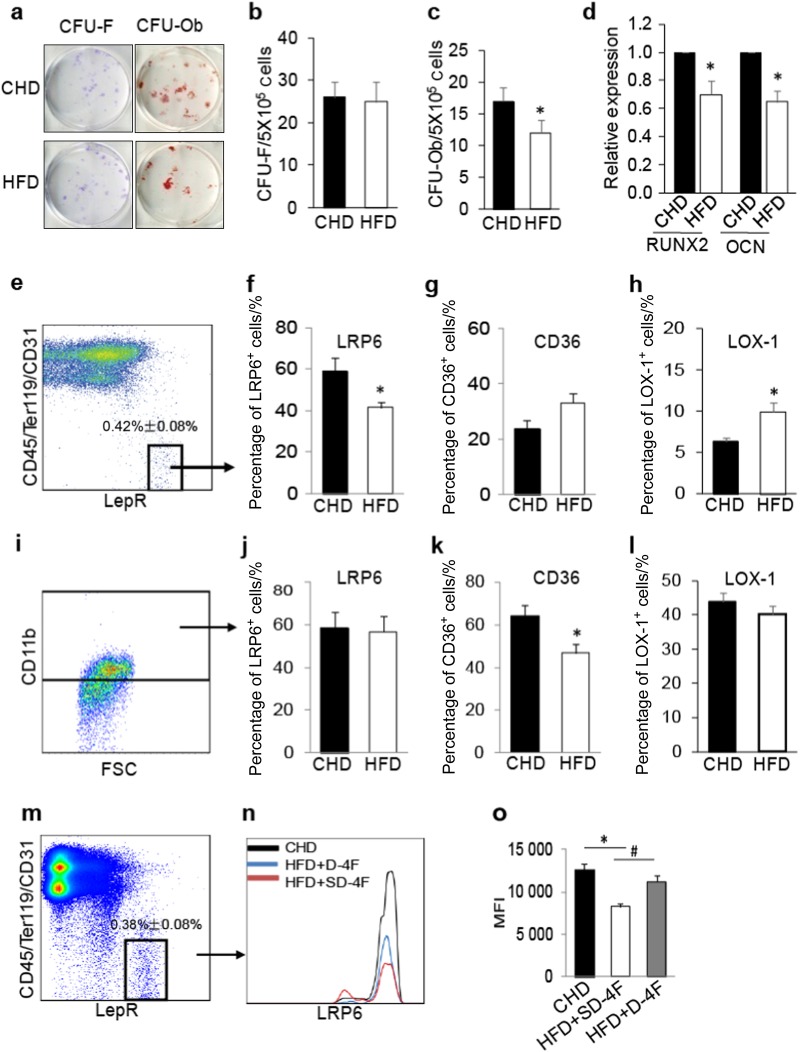
Cell Surface LRP6 Is Reduced in Bone Marrow MSCs under Microenvironment of High oxPLs. **a–c** Representative images (**a**) and the quantified CFU-F frequency (**b**), and CFU-Ob frequency (**c**) of bone marrow MSCs from 10-week-old C57BL/6 mice fed Western high fat diets (HFD) or standard chow diets (CHD) for 4 weeks. **d** Bone marrow MSCs from 10-week-old C57BL/6 mice fed HFD or CHD for 4 weeks were incubated with osteogenic medium for 7 days. mRNA expressions of RUNX2 and osteocalcin (OCN) were detected by Real-time PCR analysis. *n* = 5 mice per treatment group from six independent experiments, data are represented as mean ± s.e.m. *p < 0.05, vs. CHD group, as determined by Student’s *t*-tests. **e–h** Representative images of the flow cytometry analysis (**e**) and the percentage of LRP6^+^ (**f**), CD36^+^ cells (**g**), and LOX-1 (**h**) in LepR^+^CD45^–^CD31^–^Ter11^9-^ cell population from 10-week-old C57BL/6 mice fed Western high fat diets (HFD) or standard chow diets (CHD) for 2 weeks. *n* = 5, data are represented as mean ± s.e.m. *p < 0.01 vs. CHD group, as determined by Student’s *t*-tests. **i–l** Representative images of the flow cytometry analysis (**i**) and the percentage of LRP6^+^ (**j**), CD36^+^ cells (**k**), and LOX-1 (**l**) in CD11b^+^ cell population from 10-week-old C57BL/6 mice fed Western high fat diets (HFD) or standard chow diets (CHD) for 2 weeks. *n* = 5, data are represented as mean ± s.e.m. *p < 0.01 vs. CHD group, as determined by Student’s *t*-tests. **m–o** 10-week-old C57BL/6 mice fed CHD or HFD together with D-4F peptide (HFD + D-4F) or scrambled D-4F peptide (HFD + SD-4F) for 2 weeks. The peptides were administered orally in drinking water at 0.4 mg·mL^-1^, equivalent to 1.6 mg/d. Flow cytometry analysis of LRP6^+^ cells in bone marrow LepR^+^CD45^–^CD31^–^Ter11^9-^ MSCs (**i**). Representative histograms showing relative intensity of LRP6 antibody-conjugated fluorescence in the three groups (**j**). Quantitative analysis of the relative fluorescence intensity in the three groups (**k**). *n* = 5, data are represented as mean ± s.e.m. *p < 0.01 vs. CHD group, ^#^*p* < 0.01, vs. HFD + SD-4F group, as determined by Student’s *t*-tests

We examined whether increased oxPL production in bone marrow microenvironment leads to functional decline of bone marrow mesenchymal stromal cells (MSCs). Bone marrow MSCs from mice fed a Western high fat diet (HFD) for 4 weeks showed relatively normal CFU-F but a reduction in CFU-Ob (Fig. 1a–c) compared with the cells from the mice fed a standard chow diet (CHD). Consistently, bone marrow MSCs from HFD-fed mice had reduced expression of the osteoblast differentiation marker gene runt related transcription factor 2 (*RUNX2*) and Osteocalcin after the cells were incubated with an osteogenic medium (Fig. 1d). Thus, bone marrow MSCs in HFD-fed mice had an impaired osteogenic differentiation capacity. We then assessed the changes of LRP6 in bone marrow MSCs in HFD-fed mice. We performed flow cytometry analysis of LRP6 expression in bone marrow LepR^+^CD45^–^CD31^–^Ter119^–^ cells, which are recognized as multipotent MSCs in adult bone marrow.^36,37^ The percentage of LRP6^+^ cells was markedly decreased in the LepR^+^CD45^–^CD31^–^Ter119^–^ cell population (Fig. 1e and left panel in Fig. 1f) but not in CD11b^+^ macrophages (Fig. 1g and left panel in Fig. 1h) in bone marrow from HFD-fed mice compared with that of CHD-fed mice. CD36 and LOX-1 are known SRs of oxPLs.^38–41^ In contrast to LRP6, the percentage of CD36^+^ cells was unchanged in MSC population (right panel in Fig. 1g) but dramatically reduced in macrophages (right panel in Fig. 1k) in HFD-fed mice relative to CHD-fed mice. The percentage of LOX-1^+^ cells was elevated in bone marrow MSCs (Fig. 1h) and unchanged in macrophages (Fig. 1l) from HFD mice relative to CHD mice. ROS in bone marrow MSCs and serum level of malonaldehyde (MDA), a stable end product of lipid peroxidation, were significantly elevated in HFD-fed mice relative to CHD-fed mice (Figure S1a and S1b), indicating possible involvement of oxPLs in the reduction in osteoblast differentiation ability and LRP6 level in MSCs. We then examined whether cell surface LRP6 level in MSCs could be restored by treating the HFD mice with D-4F, an apolipoprotein A-I mimetic peptide that scavenges ROS and suppresses the production of lipid peroxide.^42,43^ Indeed, D-4F increased the percentage of LRP6^+^ cells in bone marrow LepR^+^CD45^–^CD31^–^Ter119^–^ MSCs in HFD-fed mice to a similar level as in CHD mice (Fig. 1m–o). On the contrary, SD-4F, an inactive scrambled control peptide, was unable to have such effect and failed to change the percentage of LRP6^+^ cells in MSCs from HFD-fed mice (Figure S2). The results suggest that LRP6 on the cell surface of MSCs may respond specifically to the increased oxPLs in bone marrow microenvironment.

### oxPLs induce a rapid decrease in cell surface LRP6, but not LRP5

**Fig. 2:**
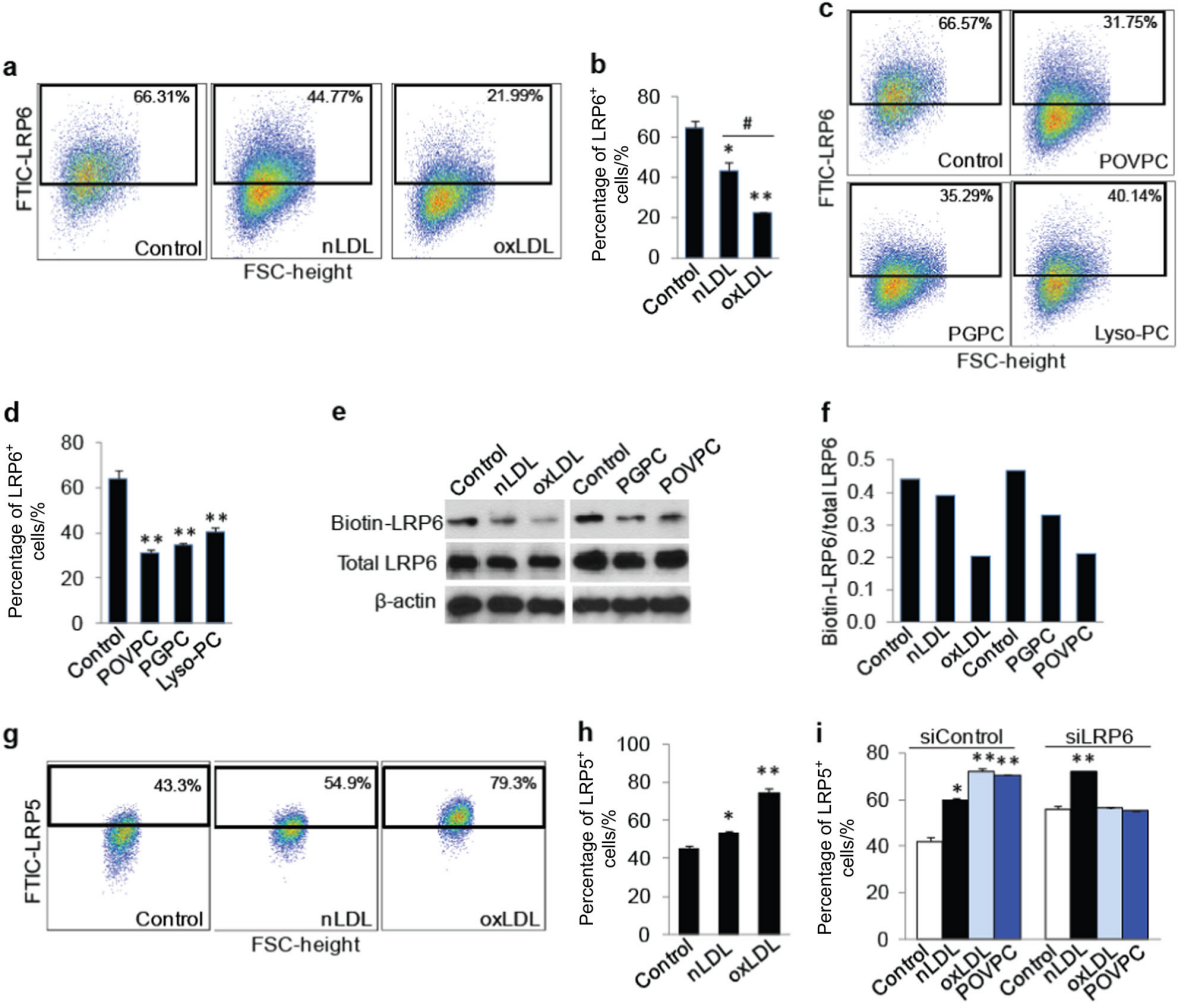
OxPLs Reduce LRP6 Expression on MSC Surface. **a**, **b** Representative images of the flow cytometry analysis (**a**) and the percentage of LRP6^+^ cells (**b**) in human MSCs treated with PBS (Control), 20 μg·mL/ml nLDL or oxLDL (dissolved in PBS) for 30 min. *p < 0.01, **p < 0.001 vs. Control group; #*p* < 0.01 vs. nLDL group, as determined by Student’s *t*-tests. **c,** **d** Representative images of the flow cytometry analysis (**c**) and the percentage of LRP6^+^ cells (**d**) in human MSCs treated with 10 μmol·L^-1^ various lipids in 1% ethanol as indicated for 30 min. Control cells were treated with 1% ethanol. *n* = 5, data are represented as mean ± s.e.m. *p < 0.01, **p < 0.001 vs. Control group; #*p* < 0.01 vs. nLDL group, as determined by Student’s *t*-tests. **e**, **f** Human MSCs were treated with various lipids as indicated for 30 min. Cell surface proteins were labeled with Sulfo-NHS-SS-Biotin. Cells were lysed, and biotinylated proteins were precipitated with streptavidin-agarose. Biotinylated and total LRP6 was evaluated by Western blot analysis (**e**). The cell surface biotinylated LRP6 was normalized by quantitation of the band density and calculation of the density ratio of the band in the 1^st^ row to the corresponding band in the 2^nd^ row in (**f**). **g**, **h** oxLDL increases LRP5 level on cell surface of MSCs. Representative images of the flow cytometry analysis (**g**) and the percentage of LRP5^+^ cells (**h**) in human MSCs treated with PBS (Control), 20 μg·mL^-1^ nLDL and oxLDL (dissolved in PBS) for 30 min. *n* = 6, data are represented as mean ± s.e.m. *p < 0.05, **p < 0.01, vs. Control group, as determined by Student’s *t*-tests. **i** Human MSCs were transfected with control siRNA or LRP6 siRNA. 2 days later, cells were treated with various lipids as indicated for 30 min. Flow cytometry analysis was performed and the percentage of LRP5^+^ cells was calculated. *n* = 6, data are represented as mean ± s.e.m. *p < 0.05, **p < 0.01, vs. Control group, as determined by Student’s *t*-tests

We then tested whether oxPLs directly affect LRP6 expression in MSCs. Treatment of bone marrow–derived MSCs with oxLDL for 30 min led to a reduced percentage of LRP6^+^ cells relative to untreated (control) cells. Moreover, the reduction in the percentage of LRP6^+^ cells was also significant in oxLDL- relative to non-oxidative nLDL-treated cells although nLDL also induced a reduction in the percentage of LRP6^+^ MSCs (Fig. 2a, b). As the activity of oxLDL is contained primarily in its lipid fraction,^44^ we tested whether individual biologically active oxPLs exert a similar effect on MSCs as does the oxLDL particle. MSCs treated with the same concentration of POVPC or PGPC, major components of oxPLs within oxLDL, both decreased the percentage of LRP6^+^ cells (Fig. 2c, d), supporting the role of POVPC and PGPC as major component oxPLs in oxLDL for its action on MSCs. The percentage of LRP6^+^ cells in MSCs was also reduced by Lyso-PC, another component of oxPLs. Consistently, MSCs treated with oxLDL, POVPC, or PGPC had reduced cell surface LRP6 expression relative to the vehicle control group. However, the expression level of LRP6 in the total cell lysates of MSCs was unchanged after the treatment of oxLDL or individual oxPLs (Fig. 2e, f). We also detected the change of cell surface LRP5, which has a high degree of sequence homology with LRP6, in MSCs after oxPLs treatment. There is an increase in the percentage of LRP5^+^ cells in nLDL- and oxLDL-treated cells relative to control cells, with the effect of oxLDL being much more profound (Fig. 2g, h). Moreover, siRNA-induced LRP6 silence in MSCs almost completely abrogated the oxLDL- and POVPC-increased LRP5 level with no significant effect on nLDL-induced LRP5 elevation on MSCs (Fig. 2i), indicating that the elevated LRP5 in response to oxPLs is a compensatory result of the reduced LRP6 level. Thus, LRP6 on cell surface of MSCs may act as a specific sensor of oxPLs within the bone marrow microenvironment.

### LRP6 binds and mediates the uptake of oxPLs by MSCs

**Fig. 3:**
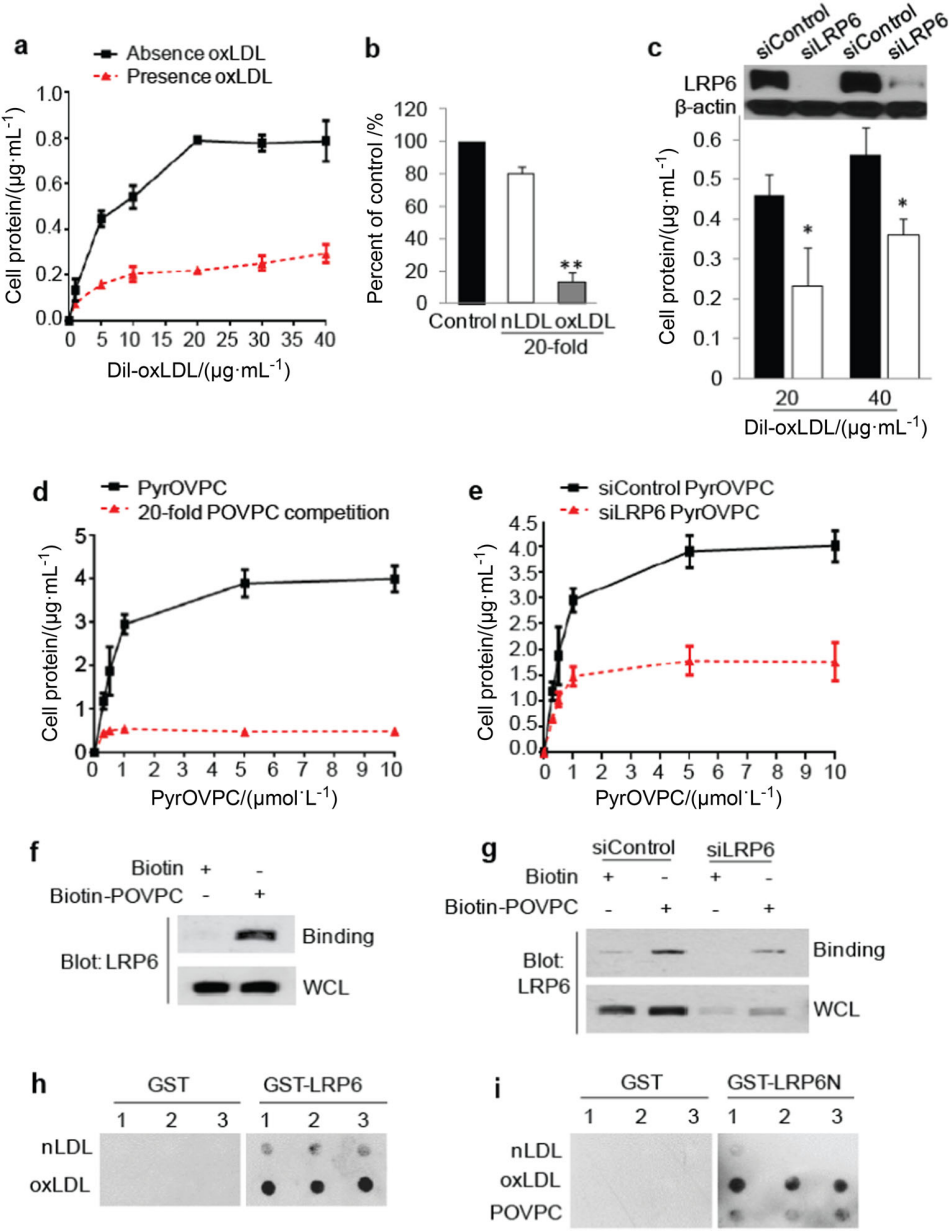
OxPLs bind to cell surface LRP6 in MSCs. **a** Dose-response relationship in DiI-oxLDL binding to MSCs. Human MSCs were incubated with increasing concentration of DiI-oxLDL (dissolved in PBS) for 2 h at 4 ℃ in the absence (black line) or presence (red line) of a 20-fold excess of unlabeled oxLDL. Binding was determined by detecting the fluorescence emission spectra as stated under Materials and Methods. **b** Human MSCs were incubated with 20 μg·mL^-1^ DiI-oxLDL in the absence (black bar) or presence of 20-fold excess of nLDL (white bar) or oxLDL (gray bar) for 2 h at 4 ℃. Binding was determined by detecting the fluorescence emission spectra. **P < 0.01 vs. control group, as determined by Student’s *t*-tests. **c** Human MSCs were transfected with either irrelevant control siRNA (siControl) or LRP6 siRNA (siLRP6). 2 days later, the cells were incubated with 20 or 40 μg·mL^-1^l DiI-oxLDL (dissolved in PBS) for 2 h at 4℃. *n* = 6, data are represented as mean ± s.e.m. *p < 0.05, **p < 0.01 vs. control group, as determined by Student’s *t*-tests. **d** Dose-response relationship in PyrOVPC binding to MSCs. Human MSCs were incubated with increasing concentration of PyrOVPC for 2 h at 4 ℃ in the absence (black line) or presence (red line) of a 20-fold excess of unlabeled POVPC. Binding was determined by detecting the fluorescence emission spectra. **e** Human MSCs were transfected with either control siRNA or LRP6 siRNA. 2 days later, the cells were incubated with increasing doses of PyrOVPC for 2 h at 4℃. *n* = 6, data are represented as mean ± s.e.m. **f** MSCs were incubated with biotin or biotin-labeled POVPC for 30 min, washed, and the cell lysates were incubated with Streptavidin-agarose beads. The precipitated proteins were subjected to western blot with LRP6 antibody. WCL, whole cell lysates. **g** MSCs were transfected with irrelevant control siRNA or LRP6 siRNA. 2 days later, the cells were treated with biotin or biotin-labeled POVPC for 30 min, washed, and the cell lysates were incubated with Streptavidin-agarose beads. The precipitated proteins were subjected to Western blot with LRP6 antibody. **h** Protein lipid overlay assays. 20 μg·mL^-1^ of nLDL or oxLDL were spotted onto nitrocellulose membranes (triplicates), which were incubated with the purified GST or GST-LRP6 protein. Protein bound to the membrane by virtue of their interaction with lipids was detected using GST antibody. **i** nLDL, oxLDL, or POVPC were spotted onto nitrocellulose membrances (triplicates), which were incubated with the purified GST or GST-LRP6N protein. Protein bound to the membrane by virtue of their interaction with lipids was detected using GST antibody

We examined whether there is a direct binding of oxPLs and LRP6. The binding of fluorescence 1,1′-dioctadecyl-3,3,3′,3′-tetramethyl-indocarbocyanine perchlorate (DiI)-labeled oxLDL (DiI-oxLDL) to the cell surface of MSCs was analyzed using fluorescence spectra. A dose-response relationship in DiI-oxLDL binding to MSCs showed saturation kinetics (Fig. 3a), and the binding was effectively blocked by 20-fold excess amounts of unlabeled oxLDL but not by the same amount of nLDL (Fig. 3a, b). Knockdown of LRP6 in MSCs significantly inhibited the binding of cell surface–bound DiI-oxLDL (Fig. 3c). Similarly, a specific binding curve of Pyrene fluorescence-labeled POVPC (PyrOVPC) to MSCs was also achieved (Fig. 3d). The binding affinity of PyrOVPC to MSCs was also markedly lower after LRP6 knockdown by siRNA in MSCs (Fig. 3e). Therefore, LRP6 is a key molecule mediating the binding of oxPLs to MSCs. The binding of POVPC to LRP6 in MSCs was also assessed by precipitation assay. MSCs were treated with biotin-labeled POVPC, a biotin precipitation of the cell lysates was performed followed by western blot with LRP6 antibody. Strong binding of biotin-labeled POVPC to LRP6 was detected in MSCs (Fig. 3f). The binding became much weaker after the knockdown of LRP6 by siRNA (Fig. 3g), indicating that POVPC specifically binds to LRP6 on MSCs. The direct binding of oxPLs to LRP6 was also detected by a protein lipid overlay (PLO) assay,^45,46^ in which the in vitro binding of the same amount of nLDL and oxLDL to full length LRP6 or the extracellular domain of LRP6 (LRP6N) was detected. oxLDL but not nLDL had a strong interaction with the purified full length LRP6 fused with GST (GST-LRP6) (Fig. 3h), whereas no binding of oxLDL to GST protein alone was detected. Consistently, oxLDL and POVPC, but not nLDL, interacted with purified LRP6 extracellular domain fused with GST (GST-LRP6N) (Fig. 3i). Therefore, oxPLs bind directly to the extracellular domain of LRP6.

**Fig. 4:**
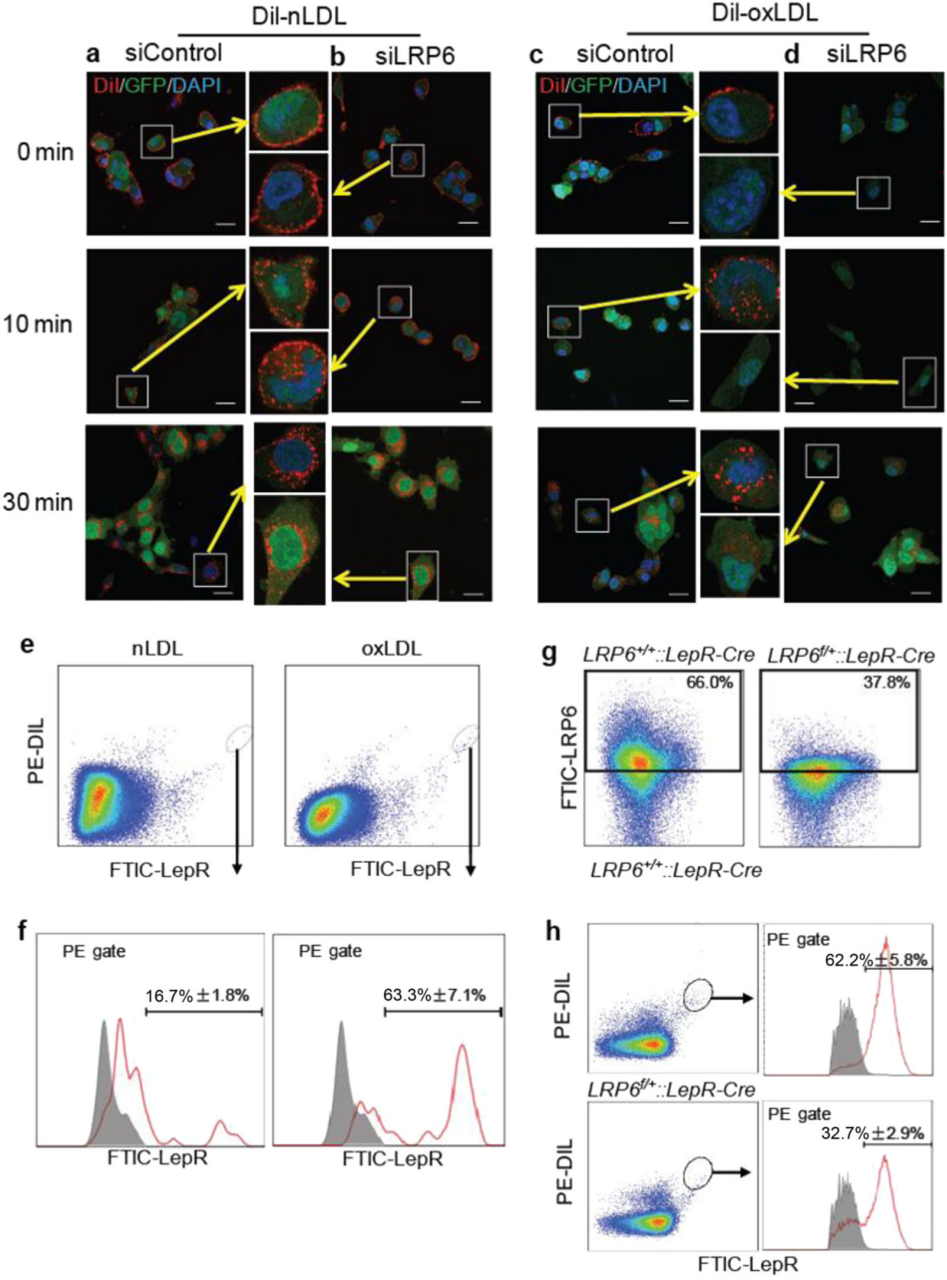
LRP6 Mediates the Uptake of oxPLs by MSCs. **a–d** Fluorescence imaging of the binding and uptake of DiI-nLDL and DiI-oxLDL (dissolved in PBS) by MSCs. Bone marrow MSCs isolated from GFP transgenic mice were transfected with either irrelevant control siRNA (siControl) (**a**, **c**) or LRP6 siRNA (siLRP6) (**b**, **d**). 2 days later, the cells were incubated with DiI-nLDL (**a,** **b**) or DiI-oxLDL (**c**, **d**) for 2 h at 4 ℃, and were then incubated at 37 ℃ for 0 min, 10 min, or 30 min. Cells were washed, fixed, and analyzed by fluorescence microscopy. (Scale bar: 10 μm). **e** Mice were injected with DiI-nLDL or DiI-oxLDL (dissolved in PBS) via tail vein at a dose of 20 μg per mouse. 4 h after injection, Mice were killed at 4 h after injection, and the bone marrow cells were subjected to flow cytometry analysis. The percentages of the DiI-nLDL or DiI-oxLDL uptake by LepR^+^CD45^–^CD31^–^Ter119^-^ MSCs are shown in the lower panel. **f**, **g** *LRP6*^*+/+*^*::LepR-Cre and LRP6*^*f/+*^*::LepR-Cre* mice were injected with DiI-oxLDL via tail vein at a dose of 20 μg per mouse. 4 h after injection, mice were killed, and bone marrow cells were subjected to flow cytometry analysis. Representative images of the percentage of LRP6^+^ cells are shown in **f**. The percentages of the DiI-oxLDL uptake by LepR^+^CD45^–^CD31^–^Ter119^-^ cells are shown in **g**. *n* = 5, data are represented as mean ± s.e.m

The direct binding of oxPLs to LRP6 and the reduced cell surface LRP6 expression in response to oxPLs indicate that LRP6 may be a specific receptor mediating oxPL uptake by bone marrow MSCs. To test this hypothesis, we used bone marrow MSCs isolated from GFP transgenic mice. Cells were treated with DiI-labeled native LDL (DiI-nLDL) or DiI-oxLDL, and the internalization of red fluorescence was detected in MSCs carrying green fluorescence. Cell surface binding of both DiI-nLDL and DiI-oxLDL were observed immediately after the treatment (0 min), and a gradual uptake of DiI-nLDL and DiI-oxLDL by MSCs (10 and 30 min) was observed in MSCs (Fig. 4a, c). Both the cell surface binding and uptake of DiI-oxLDL by MSCs were reduced dramatically in MSCs with LRP6 knockdown by siRNA (Fig. 4d), whereas the cell surface binding and uptake of DiI-nLDL by MSCs were barely changed in the cells with siLRP6 transfection relative to control siRNA-transfected cells (Fig. 4b). On the contrary, while the uptake of DiI-nLDL by MSCs were reduced in MSCs with LRP5 knockdown (Figure S2a and S2b), the uptake of DiI-oxLDL by MSCs were not affected in the cells transfected with siLRP5 (Figure S2c and S2d).

To examine the role of LRP6 in regulating oxLDL uptake by bone marrow MSCs in vivo, mice were injected with DiI-nLDL or DiI-oxLDL intravenously. The uptake of the fluorescence-labeled lipids was assessed in bone marrow LepR^+^CD45^–^CD31^–^Ter119^–^ MSCs (Figure S3). DiI-oxLDL injection resulted in obvious red fluorescence signal in bone marrow MSCs, indicating binding and/or uptake of oxLDL by this population (Fig. 4e). Whereas only 16.7% ± 1.8% of total DiI-nLDL uptake was detected in bone marrow MSCs, 63.3% ± 7.1% of DiI-oxLDL uptake was detected in this population (Fig. 4f). We then tested whether decreased LRP6 expression would result in reduced DiI-oxLDL uptake in MSCs by using *LepR-Cre; LRP6*^*+/-*^ mice, in which one allele of LRP6 is deleted in LepR^+^ cells. Flow cytometry analysis showed that the percentage of LRP6^+^ cells within bone marrow CD45^−^cells decreased nearly half in *LepR-Cre; LRP6*^*+/-*^ mice relative to *LepR-Cre; LRP6*^*+/+*^ control mice (Fig. 4g). Importantly, the percentage of DiI-oxLDL uptake by bone marrow MSCs was also reduced by nearly half after heterogeneous LRP6 knockout (Fig. 4h). Thus, LRP6 specifically mediates oxLDL uptake by bone marrow MSCs.

### oxPLs induce LRP6 endocytosis in MSCs

**Fig. 5:**
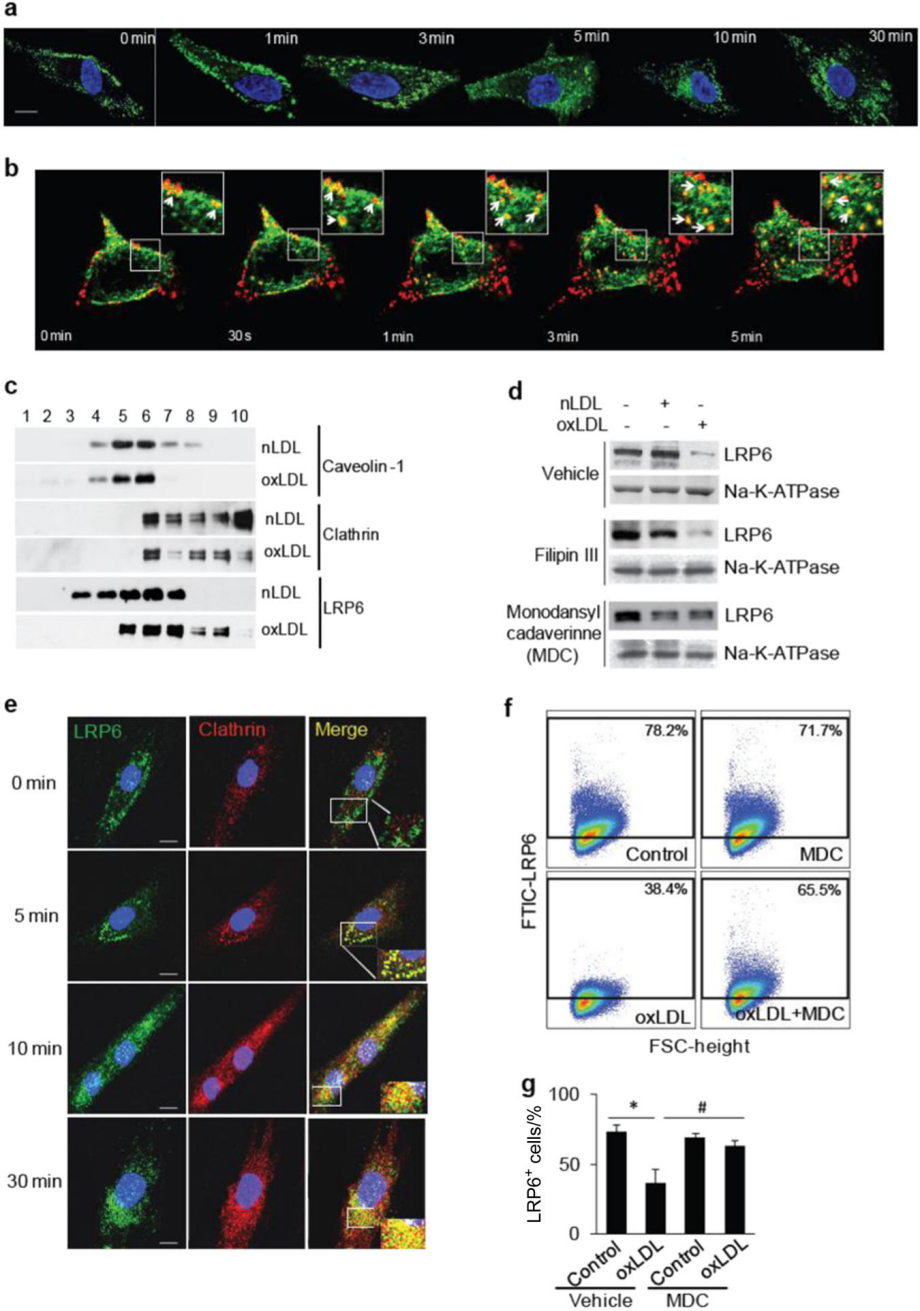
OxPLs induce LRP6 endocytosis in MSCs through clathrin-mediated pathway. **a** Localization of LRP6 in MSCs at 0, 1, 3, 5, 10, and 30 min after oxLDL treatment detected by immunofluorescence staining of LRP6 (in green). (Scale bar: 20 μm). **b** Time-lapse imaging of co-endocytosis of LRP6-eGFP and DiI-oxLDL. 293 T cells transfected with LRP6-eGFP were incubated with DiI-oxLDL for 2 h at 4 °C, and the cells were placed into the stage chamber at 37 °C and time-lapse imaging were examined with 30-s intervals. Images of merged LRP6-eGFP (green) and DiI-oxLDL (red) are presented. Insets are high magnification images of the region indicated by white rectangles, White arrows point on the examples of colocalization of LRP6-eGFP and DiI-oxLDL. (Scale bars: 20 μm). **c** Redistribution of LRP6 between the lipid raft and nonlipid raft fractions in response to oxLDL. MSCs were treated with 20 μg/ml nLDL or oxLDL for 30 min. Cell lysates were fractionated by sucrose density gradient centrifugation, and aliquots were probed with anti-LRP6. Caveolin-1 and clathrin indicate the positions of the lipid raft and nonlipid raft fractions, respectively. **d** MSCs were incubated with 20 μg·mL^-1^l nLDL or oxLDL together with DMSO (vehicle), Filipin III (3 μg·mL^-1^ in DMSO) or MDC (100 μmol·L^-1^" in DMSO) for 30 min. Cell surface proteins were labeled with Sulfo-NHS-SS-Biotin. Cells were lysed, and biotinylated proteins were precipitated with streptavidin-agarose. Presence of LRP6 and Na,K-ATPase was evaluated by Western blot analysis. **e** Co-localization of LRP6 and clathrin in MSCs at 0, 10, and 30 min after POVPC treatment detected by immunofluorescence staining of LRP6 (in green) and clathrin (in red). DAPI was used to stain the nuclei.(Scale bar: 20μm). **f**, **g** Representative images of the flow cytometry analysis (**f**) and the percentage of LRP6^+^ cells (**g**) in human MSCs treated with vehicle (Control) or oxLDL in the absence or presence of MDC for 30 min. *n* = 6, data are represented as mean ± s.e.m. *P < 0.01, vs. Control group; ^#^*P* < 0.01, vs. Vehicle + oxLDL group, as determined by Student’s *t*-tests

We then tested whether LRP6 undergoes endocytosis in MSCs in the presence of oxPLs. MSCs were incubated with POVPC for different time periods, and the localization of LRP6 was analyzed visually by confocal microscopy. LRP6 resided mainly at the cell surface of MSCs immediately after oxLDL treatment (0 min), with a small portion of LRP6 in cellular plasma. At 1, 3, 5, 10, and 30 min after oxLDL treatment, LRP6 protein was gradually internalized as punctuated organelles in the cytoplasm (Fig. 5a). We also examined the co-endocytosis of LRP6 and oxLDL in living cells, in which LRP6-eGFP was overexpressed, by time-lapse imaging. Treatment of DiI-oxLDL into the microscope chamber resulted in rapid binding of DiI-oxLDL to the cell membrane and was highly co-localized with LRP6-eGFP (Fig. 5b, 0 min). A rapid movement of the DiI-oxLDL/ LRP6-eGFP cluster from cell membrane to the intracellular compartments was seen in most of the cellular regions 30 s to 5 min time period (Fig. 5b, 30′ to 5 min, white arrows indicate the DiI-oxLDL/ LRP6-eGFP cluster). In some regions of the cells only DiI-oxLDL was seen without associated LRP6-eGFP, indicating that LRP6 is not the only receptor mediating oxLDL uptake. It is known that LRP6 is a lipid raft receptor protein that is internalized after Wnt stimulation in a caveolin-mediated process.^47^ Conversely, the endocytosis of LDLR after binding to LDL is mediated by clathrin-coated vesicles.^48^ LRP6 is a member of the LDLR family, with structural domains similar to LDLR. To examine whether oxPL-induced LRP6 endocytosis occurs through caveolin- or clathrin-mediated pathways, we first fractionated the lipid raft and nonlipid raft fractions of the cells treated with nLDL or oxLDL by sucrose density gradient centrifugation. The detergent-resistant membranes, including caveolin-1, were obtained in lower-density fractions (Fig. 5c, fractions 4–6 in the upper two panels), whereas the soluble membranes, including clathrin, were in higher-density fractions (Fig. 5c, fractions 6–10 in the middle two panels). LRP6 was detected mainly in the caveolin-associated lipid raft fraction in nLDL-treated cells (Fig. 5c, fraction 3–7 in the fifth panel). After oxLDL treatment, a reduced proportion of LRP6 was present with caveolin but an increased proportion of LRP6 was present with clathrin (Fig. 5c, fraction 5−9 in the sixth panel). Therefore, oxLDL may induce LRP6 endocytosis through a clathrin-associated pathway. We then assessed the effects of pharmacological inhibitors of clathrin-mediated and caveolin-mediated endocytosis on LRP6 expression on the MSC surface after the cells were stimulated with nLDL or oxLDL. MSCs incubated with oxLDL but not nLDL had significantly reduced LRP6 expression on cell surface (Fig. 5d). Importantly, oxLDL-induced cell surface LRP6 reduction was antagonized by clathrin-dependent endocytosis inhibitor monodansylcadaverine (MDC) but not by Filipin III, an inhibitor of caveolin-mediated endocytosis (Fig. 5d), indicating that oxPL-induced endocytosis of LRP6 is dependent on clathrin- but not caveolin-mediated pathway. Consistently, immunofluorescence co-localization assays showed no co-localization between LRP6 and clathrin in MSCs in the absence of POVPC treatment (Fig. 5e, first row). At 5 min after POVPC treatment, co-localization of LRP6 and clathrin was detected and the complex was retained mostly at the cell surface (Fig. 5e, second Row). At 10 and 30 min after POVPC treatment, the LRP6/clathrin co-localization was mainly detected as cytoplasmic puncta (Fig. 5e, third and fourth Row). Flow cytometry analysis of cell surface LRP6 expression further confirmed the effect of MDC on the restoration of the cell surface LRP6 expression that was reduced by oxLDL (Fig. 5f, g).

### oxPLs impair LRP6-mediated signaling pathways in bone marrow MSCs

**Fig. 6:**
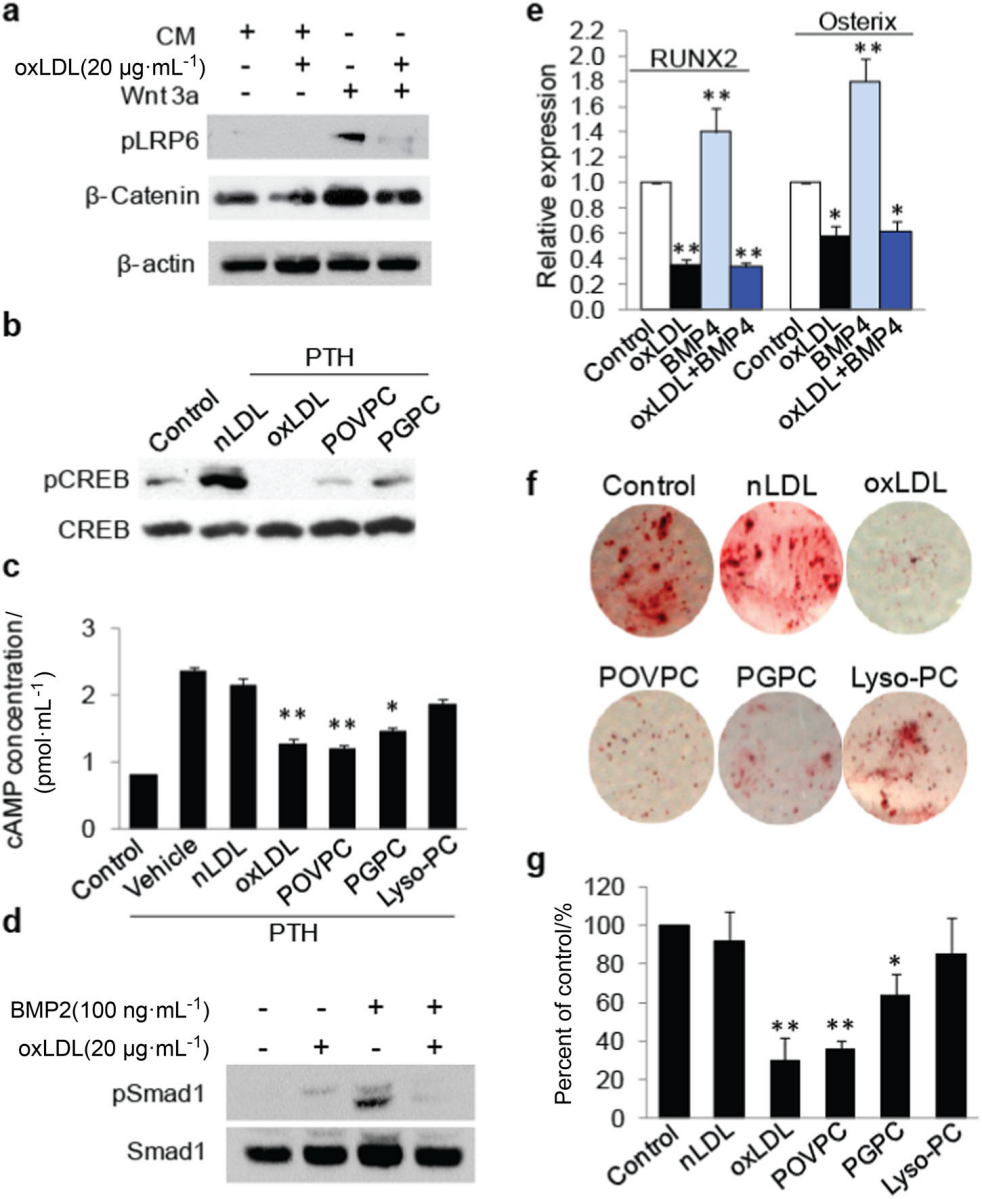
OxPLs impair LRP6-mediated signaling pathways in bone marrow MSCs. **a** Human bone marrow MSCs were treated with control conditioned medium or Wnt3a conditioned medium containing vehicle or oxLDL as indicated. Protein extract of the cells was subjected to western blot using antibodies to phosphorylated LRP6 (pLRP6), β-catenin or β-actin. **b** MSCs were treated with vehicle or 50 nmol·L^-1^ PTH together with individual lipids as indicated (nLDL and oxLDL were 20 μg·mL^-1^; POVPC and PGPC were 10 μmol·L^-1^) for 1 h. Protein extracts of the cells were subjected to Western blot using antibodies to phosphorylated CREB or total CREB. **c** MSCs were treated with vehicle or 50 nM PTH together with individual lipids as indicated (nLDL and oxLDL were 20 μg·mL^-1^l; POVPC, PGPC, and LysoPC were 10 μmol·L^-1^) for 1 h. cAMP produced by the cells was detected. *n* = 6, data are represented as mean ± s.e.m. *P< 0.05, **P < 0.01 vs. PTH + Vehicle group, as determined by Student’s *t*-tests. **d**, **e** Human bone marrow MSCs were treated with BMP2 and/or 20 µg·mL^-1^ oxLDL as indicated. Protein extract of the cells was subjected to western blot using antibodies to pSmad1 or total Smad1 (**d**). RT-qPCR analysis of RUNX2 and osterix mRNA in MSCs treated with 50 ng·mL^-1^l BMP4 and/or 20 µg·mL^-1^l oxLDL as indicated (**e**). **f**, **g** MSCs were cultured in osteogenic medium with individual lipids as indicated (nLDL and oxLDL were 20 μg·mL^-1^; POVPC and PGPC were 10 μmol·L^-^^1^). Cells were fixed and stained with Alizarin red S 21 days after treatment. Representative images were shown in **f**. Relative intensity of the Alizarin red staining in each group was normalized to the level of control group (**g**)

LRP6 mediates the signaling pathways of Wnts, PTH, and BMPs, which are factors known to stimulate the osteoblast differentiation of MSCs.^5–7,9,49^ We examined the response of MSCs to these osteogenic factors with or without the treatment of oxLDL or individual oxPLs. oxLDL almost completely abolished Wnt3a-stimulated LRP6 phosphorylation and β-catenin stabilization (Fig. 6a). Similarly, oxLDL but not nLDL abolished PTH-stimulated cAMP-response element binding protein (CREB) phosphorylation (Fig. 6b) and cAMP production (Fig. 6c). Consistent with the impaired osteoblast differentiation capacity of MSCs, PTH-elicited CREB phosphorylation and cAMP production were also dramatically inhibited by POVPC and PGPC (Fig. 6b, c). oxLDL also had significant adverse effects on BMP2-stimulated Smad1 phosphorylation (Fig. 6d) and BMP4-induced RUNX2 and osterix expression in MSCs (Fig. 6e). Moreover, osteoblast differentiation capacity of bone marrow-derived MSCs was almost abolished by oxLDL, POVPC, and PGPC, as indicated by Alizarin red staining of the osteogenic medium-incubated cells (Fig. 6f, g). nLDL did not show a significant effect on the differentiation capacity of MSCs, and Lyso-PC only moderately inhibited the differentiation of MSCs. Therefore, LRP6 is a major molecular target for the adverse effect of oxPLs on MSCs for their osteoblast differentiation ability.

**Fig. 7:**
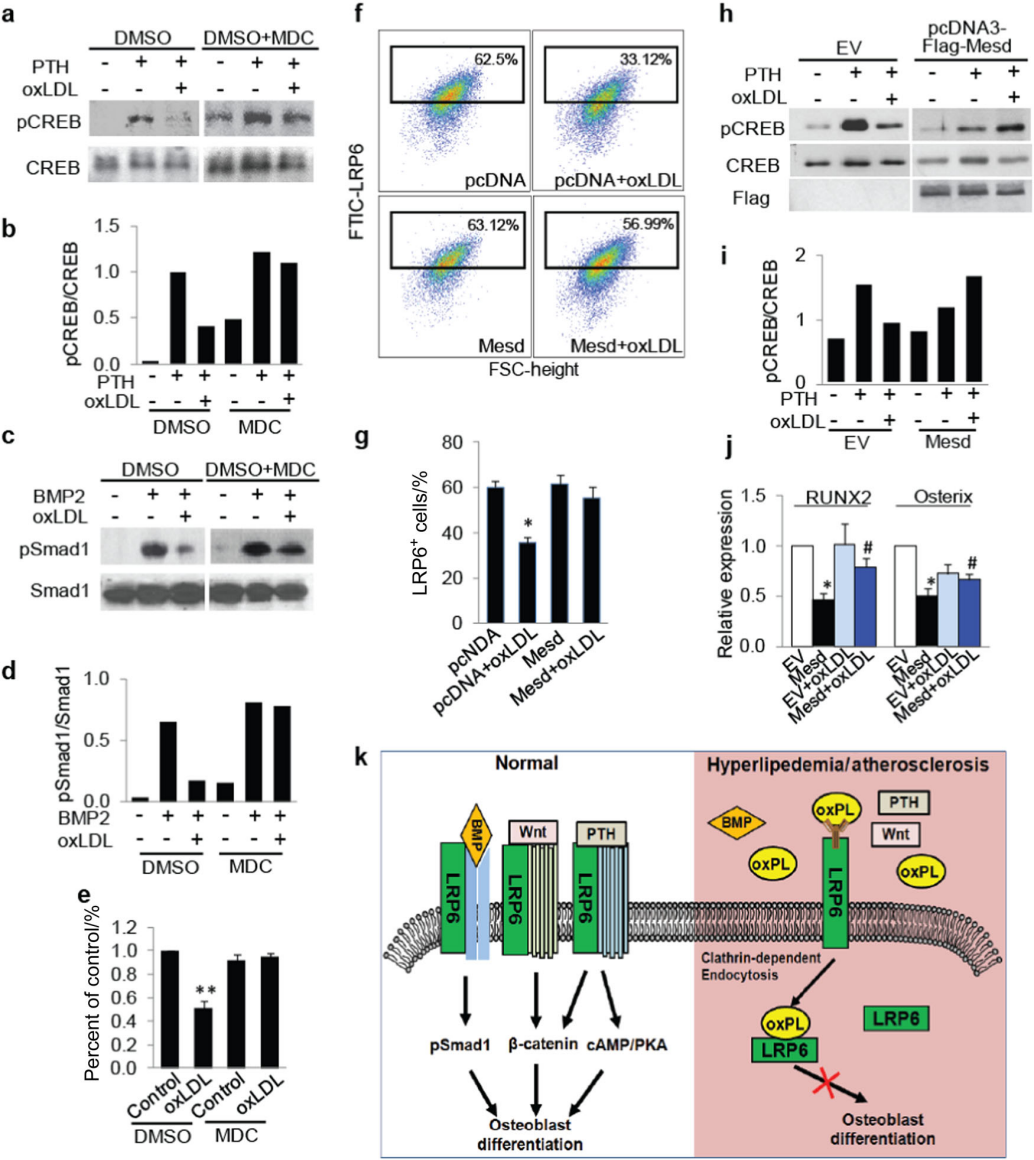
Manipulating cell surface LRP6 level antagonizes the adverse effect of oxPLs on MSCs. **a**, **b** MSCs were treated with vehicle or 50 nmol·L^-1^ PTH in the absence or presence of 20 μg·mL^-1^l oxLDL and/or 100 μmol·L^-1^ MDC for 1 h. Protein extracts of the cells were subjected to Western blot using antibodies to phosphorylated CREB or total CREB (**a**). Relative quantification for phosphor-CREB expression normalized with respect to total CREB (**b**). **c**, **d** MSCs were treated with 50 ng·mL^-1^ BMP2 in the absence or presence of 20 μg·mL^-1^ oxLDL and/or 100 μmol·L^-1^ MDC for 1 h. Protein extracts of the cells were subjected to Western blot using antibodies to phosphorylated Smad1 or total Smad1 (**c**). Relative quantification for phosphor-Smad1 expression normalized with respect to total Smad1 (**d**). **e** MSCs were cultured in osteogenic medium with 20 μg/ml oxLDL and/or 100 μmol·L^-1^ MDC. Cells were fixed and stained with Alizarin Red S 21 days after treatment. Relative intensity of the Alizarin Re·d staining in each group was normalized to the level of control group. **f**, **g** MSCs were transfected with pcDNA3 or pcDNA3-Mesd plasmids and treated with 20 μg·mL^-1^ oxLDL for 30 min. Representative images of the flow cytometry analysis (**f**) and the percentage of LRP6^+^ cells (**g**). *n* = 5, data are represented as mean ± s.e.m. *P < 0.05, vs. pcDNA group; ^#^*P* < 0.05, vs. pcDNA + oxLDL group, as determined by Student’s *t*-tests. **h**, **i** MSCs were transfected with pcDNA3 (EV) or pcDNA3-Mesd plasmids and treated with 50 nmol·L^-1^ PTH and/or 20 μg/ml oxLDL for 1 h. Protein extracts of the cells were subjected to Western blot using antibodies to phosphorylated CREB or total CREB (**h**). Relative quantification for phosphor-CREB express·ion normalized with respect to total CREB (**i**). **j** MSCs were transfected with pcDNA3 (EV) or pcDNA3-Mesd plasmids and incubated with osteogenic medium with or without 20 μg·mL^-1^ oxLDL for 7 days. mRNA expressions of RUNX2 and osterix were detected by Real-time PCR analysis. *n* = 5, data are represented as mean ± s.e.m. *P < 0.01 vs. EV group; ^#^p < 0.05 vs. EV + oxLDL group, as determined by Student’s *t*-tests. **k** Schematic model illustrating the role of LRP6 in oxPLs-induced MSC functional loss. Cell surface LRP6 in MSCs can be recruited by the receptors of Wnts, PTH, and BMPs and mediates their signaling activation for osteoblast differentiation (left panel). During hyperlipidemia and atherosclerosis, high level of oxPLs in bone marrow directly bind cell surface LRP6 and induce rapid LRP6 endocytosis, leading to blunted responses of MSCs to these osteogenic factors (right panel)

If oxPL-induced LRP6 endocytosis is a primary contributor to the diminished osteogenic potential of MSCs and declined response of the cells to osteogenic factors, inhibition of LRP6 endocytosis or increased cell surface LRP6 expression would antagonize the adverse effect of oxPLs on MSCs. To test this hypothesis, we first assessed the effect of the clathrin-dependent endocytosis inhibitor MDC on the functional changes of oxLDL-treated MSCs. Consistent with Fig. 6b, c, oxLDL almost abolished PTH-stimulated CREB phosphorylation (Fig. 7a, b) and BMP-stimulated Smad1 phosphorylation in MSCs (Fig. 7c, d), respectively. Importantly, co-treatment of the cells with MDC almost restored the signaling activation elicited by these osteogenic factors. Furthermore, osteoblast differentiation capacity of the cells co-treated with oxLDL and MDC was significantly augmented compared with those treated with oxLDL alone, as detected by Alizarin red staining (Fig. 7e). Mesoderm Development (Mesd), an ER chaperone protein, has been reported to be required for the trafficking of LRP5/6 to the cell surface.^50,51^ We examined whether overexpression of Mesd would restore the osteoblastic differentiation potential of MSCs and their response to osteogenic factors. As we expected, MSCs transfected with Mesd completely restored oxLDL-reduced cell surface LRP6 expression (Fig. 7f, g). Accordingly, Mesd overexpression also restored PTH-stimulated CREB phosphorylation (Fig. 7h, i), and the expression of osteoblast differentiation marker genes (Fig. 7j) in MSCs, which were impaired by oxLDL treatment. Taken together, these results suggest that increasing the level of cell surface LRP6 restores the blunted response of MSCs to osteogenic factors and the osteoblast differentiation capacity of MSCs caused by oxPLs.

## Discussion

Tissue culture and animal studies of the past two decades suggest a direct action of oxPLs on bone cells.^31–33^ Specifically, individual oxPLs and oxLDL inhibited the differentiation of osteoblasts and promoted the differentiation of osteoclasts. These bioactive lipids attenuate the effects of several osteogenic factors such as BMPs, Wnt, and PTH.^32,43,52^ oxPLs also play a role in directing the differentiation of bone marrow MSCs away from osteoblastic lineage and toward adipogenic lineage. Our results from this study suggest that LRP6 serves as a specific receptor for oxPLs and is a major molecular target of oxPLs for their negative actions on bone marrow MSCs (Fig. 7k). Evidence to support this concept came from flow cytometry analysis, fluorescence spectra-based ligand-receptor binding assays, biotin-precipitation assays, PLO assays, and subcellular localization assays showing a specific binding of LRP6 with oxPLs, followed by LRP6 endocytosis. We showed that LRP6 on the cell surface of bone marrow MSCs was reduced under the increased oxPLs in bone marrow microenvironment in mice. Direct treatment of the MSCs with oxPLs also induced rapid reduction in cell surface LRP6. Therefore, the in vivo and in vitro results implicate that LRP6 on MSC surface senses and responds to the change in oxPLs in the extracellular environment. Several SRs of oxLDL, such as CD36, LOX-1, SR-AI, SR-BI, and TLR-4, have been identified.^30^ These receptors directly bind oxPLs and mediate the uptake of oxLDL and oxPLs. Specifically, CD36 and SR-A are principal SRs accounting for up to 90% of oxLDL loading in macrophages,^26^ which are key players in all stages of atherosclerosis. The fact that cell surface CD36 was unchanged in bone marrow MSCs but reduced in macrophages in response to a high level oxPLs suggests that CD36 serves as a receptor of oxPLs in macrophages, and LRP6 may function as a specific oxPL receptor in MSCs. LRP6 may not be the only oxPLs receptor in MSCs as we found that the binding of POVPC to MSC cell surface was not fully blocked by LRP6 knockdown in the binding assays. The expression of LOX-1 was elevated in bone marrow MSCs from HFD mice relative to CHD mice, suggesting an involvement of LOX-1 in oxPL-induced functional change in bone marrow MSCs. It remains to be determined whether LOX-1 serves as another receptor for oxPLs in MSCs and its distinct role in regulating MSC functions. Previous studies showed that LRP6 functions as a receptor for nLDL clearance in splenic B-lymphocytes and fibroblasts.^53,54^ nLDL and oxLDL act on different receptors for their uptake by cells, and it is likely that the same nLDL or oxLDL uses distinct receptors in different types of cells in a tissue-specific manner.

An interesting phenomenon from the present study is the apparently different response of LRP5 and LRP6 in MSCs to oxPL stimulation. In contrast to the reduced cell surface LRP6 in MSCs with oxLDL- and individual oxPL-treatment, LRP5 cell surface level was significantly increased in both nLDL- and oxLDL-treated cells. oxLDL-induced, but not nLDL-induced, LRP5 elevation was completely abolished by LRP6 knockdown in MSCs, indicating a LRP6-dependent effect. These results suggest that oxPLs-induced increase in cell surface LRP5, which shares 71% amino-acid homology to LRP6,^55,56^ is a compensatory effect of oxPL-induced LRP6 reduction in MSC surface. The possible reason for the blunted Wnt/β-catenin signaling in response to oxPL stimulation is that the increased LRP5 level is still unable to fully compensate the impaired WNT/β-Catenin signaling caused by LRP6 deficiency. Increasing evidence suggest that although both LRP5 and 6 serve as Wnt co-recepters for the canonical β-catenin signaling, LRP6 is more effective than LRP5 in transducing the Wnt signaling. Genetically, the developmental phenotypes of *Lrp6* mutant mice is much more severe than LRP5 mutant.^2,34,57^ Functionally, expression of LRP6 alone, but not LRP5 alone, is able to induce a secondary axis in *Xenopus* embryos.^1^ Similarly in mammalian cell culture, LRP5 only weakly activates the Wnt pathway in the absence of an exogenous Wnt, in contrast to the highly active LRP6.^1,58^ Structurally, LRP6 cytoplasmic domain harbors stronger signaling activity than that of LRP5.^59^ Therefore, it is possible that LRP5 level was elevated in response to LRP6 deficiency as a compensatory mechanism; however, it failed to completely restore WNT/β-Catenin signaling. We also noticed that in the LRP5 knockdown MSCs, nLDL uptake, but not oxLDL uptake was significantly reduced relative to control cells. Therefore, although LRP5 and LRP6 are highly homologous and they both possess the capacity to bind lipoproteins,^53,54,60^ they exert distinct actions on MSCs in regulating lipid homeostasis/metabolism. LRP6 is a key molecule in transducing the effects of oxidized lipoprotein and oxidized lipids, whereas LRP5 appears to regulate native oxidized lipoprotein or lipid uptake by MSCs or other bone cells. Consistent with this finding, an earlier study revealed that LRP5, but not LRP6, regulates fatty acid utilization by osteoblasts.^61^

We show that MSCs are a major cellular target of oxPLs in bone marrow in vivo. It has been recognized that LepR^+^ MSCs include nearly all of the CFU-F, and they are the major source of bone-forming osteoblasts in adult bone marrow.^36,37^ We found that most of the injected Dil-oxLDL (>60%) was taken up by the bone marrow LepR^+^CD45^–^CD31^–^Ter119^-^ MSC population. In adult bone marrow, MSCs are often located by sinusoids as perivascular cells, and thus have greater exposure to bioactive oxPLs compared with other bone cells, such as mature osteoblasts and osteoclasts. Our finding implicates that bone marrow MSCs are likely one of the most deteriorated cells in pathological conditions such as hyperlipidemia, atherosclerosis, and inflammatory diseases. We also found that bone marrow MSCs treated with oxPLs had diminished osteoblast differentiation capacity, further suggesting that diminished osteogenesis by MSCs is a key player in hyperlipidemia/atherosclerosis-induced bone loss. It has been recognized that bioactive oxPLs attenuate the effects of several osteogenic factors, such as BMPs, Wnt, and PTH in mice and humans with hyperlipidemia.^32,43,52^ Consistent with these observations, we detected inhibited signaling transduction of PTH, Wnt, and BMP after treatment with oxPLs. Furthermore, we identify oxPL-LRP6 binding and the resultant LRP6 endocytosis in MSCs as a primary mechanism underlying the adverse effects of oxPLs on MSCs. LRP6 is a positive regulator of osteoblastic bone formation^7,34,35^ and is required for signaling activation elicited by several important osteogenic factors, including Wnt, PTH, PDGF, and BMP. Moreover, intermittent injection of synthetic PTH1-34 (teriparatide) is a FDA-approved anabolic therapy for osteoporosis. A key PTH cell signaling mechanism that promotes osteoblast bone formation is through interactions with LRP6.^6–12^ This study reveals that the unavailability of LRP6 on the plasma membrane of MSCs caused by oxPL-stimulated LRP6 endocytosis is one of the primary contributors to the blunted response of the cells to osteogenic factors. The fact that endocytosis inhibitor or Mesd overexpression antagonized these adverse effects of oxPLs further supports this concept and implies that manipulation of cell surface LRP6 is a potential strategy for the treatment of osteoporosis.

## Materials and Methods

### Mice and treatment

C57BL/6 mice were purchased from Jackson Lab (Bar Harbor, ME). *LRP6*^*f/f*^ mice were obtained from Van Andel Research Institute.^62,63^ Transgenic mice expressing the Cre recombinase under the control of mouse leptin receptor promoter (*LepR*-*Cre*) were purchased from Jackson Lab (stock no. 008320). Homozygous *LRP6*^*f/f*^ mice were crossed with *LepR-Cre* transgenic mice to generate heterozygous *LepR-Cre*^*+/−*^*; LRP6*^f/+^ mice. Male mice were used in all experiments. All animals were maintained in the Animal Facility of the Johns Hopkins University School of Medicine. The experimental protocol was approved by the Institutional Animal Care and Use Committee of the Johns Hopkins University, Baltimore, MD. Genomic DNA extraction and genotyping of the mice were performed as described previously.^7^

10-week-old male C57BL/6 mice were fed with CHD or HFD (referred to as “Western” diet, Harlan Teklad) for 4 weeks to detect changes in osteoblast differentiation ability of bone marrow MSCs in HFD-fed mice. 10-week-old C57BL/6 mice were fed with CHD or HFD for 2 weeks to detect changes in LRP6 cell surface expression in bone marrow MSCs. HFD- or CHD-fed mice were given 0.3 mg·mL^-1^ D-4F or scramble peptide (Biomatik) in drinking water for 2 weeks to examine the effect of D-4F peptide on changing MSC surface LRP6 level. For in vivo LDL-uptake detection, mice were injected with DiI-oxLDL or DiI-nLDL (Alfa Aesar), 20 μg per mouse, through tail vein. The time course (2 weeks treatment) and dosage (0.3 mg·mL^-1^) for D-4F peptide in the mouse study were chosen based on previous publications,^42^ in which oral administration of 0.05–1 mg·mL^-1^ D-4F peptide dramatically reduces atherosclerosis in mice.

### Flow cytometry identification of murine bone marrow MSCs

Murine bone marrow MSCs were identified and isolated using flow cytometry analyses and FACS sorting as described previously.^36,64^ At the time of euthanasia, bone marrow cells were flushed from femoral and tibial medullary cavities. Cell numbers were determined after removal of red blood cells with ammonium chloride–potassium lysis buffer (Quality Biological). Non-permeabilized cells were incubated with the antibodies and fixed in 1% paraformaldehyde. The antibodies used for flow cytometry identification of MSCs included anti-CD45-APC (Biolegend, 103112, 1:200), anti-Ter119-APC (Biolegend, 116212, 1:200), anti-CD31-APC (Biolegend, 102510, 1:100), and anti-LepR-biotin (R&D Systems, AF497, 1:500). Cells were stained with antibodies in staining buffer (HBSS + 2% fetal bovine serum) on ice for 1 h, and then washed with staining buffer. Biotin-conjugated antibody was incubated with streptavidin-brilliant violet 421 (Biolegend, 405225, 1:500) for another 20 min. LepR^+^CD45^–^CD31^–^Ter119^-^ cells was analyzed using a BD LSR II flow cytometer or sorted using a BD FACSAria IIu cell sorter.

### CFU-F, CFU-Ob, and in vitro differentiation assays of mouse bone marrow MSCs

CFU-F and CFU-Ob assays of unfractionated bone marrow cells were conducted as described^36,37^ with modification. For CFU-F assays, single-cell suspensions of bone marrow cells were prepared and plated at a density of 5 × 10^5^ cells per well in 6-well plates in DMEM (Gibco) supplemented with 20% FBS (Atlanta Biologicals), 10 μmol·L^-1^ Y-27632 (StemCell Technologies), and 1% penicillin/streptomycin (Sigma-Aldrich). The cultures were incubated at 37℃. CFU-F colonies were counted after 10 days of culture with Crystal violet staining. For the in vitro osteoblast differentiation assays, bone marrow cells were plated at a density of 5 × 10^5^ cells per well in 6-well plates in DMEM supplemented with 20% FBS, 10 μmol·L^-1^ Y-27632, and 1% penicillin/streptomycin for 7 days followed by 21 days of osteogenic differentiation with StemPro Differentiation Kits (Invitrogen). Osteogenic differentiation was detected by Alizarin red staining. The colony-forming efficiency was determined by counting the number of colonies per 5 × 10^5^ marrow cells plated.

In vitro differentiation of clonal MSCs was performed as previously described, with modification.^37^ LepR^+^CD45^–^CD31^–^Ter119^-^ MSCs were sorted into 6-well plates (800 cells per well). After 7 days, primary CFU-Fs were formed. Osteogenic differentiation was then induced by replacing the medium with StemPro Differentiation Kits (Invitrogen). Osteogenic differentiation was detected by Alizarin red staining solution after 21 days of incubation.

### Human bone marrow MSC culture and in vitro osteoblast differentiation

Human bone marrow-derived MSCs with certified MSC surface markers (Lonza) were maintained as adherent cultures in complete MSC growth medium (MSCGM BulletKit; Lonza) at 37 °C/5% CO_2_. The cells were used at early passage (<5 passages) for all experiments. For osteogenic differentiation, cells were seeded at a density of 5 × 10^3^ per cm^2^ with StemPro Osteogenesis Differentiation Kit (Invitrogen). Histochemical staining for alkaline phosphatase (ALP) activity in the cells was performed using Fast BCIP/NBT Tablets (Sigma-Aldrich) on day 14. Alizarin red staining for calcium deposits was performed on day 21.

### Flow cytometry analysis of cell surface LRP6 expression in murine and human MSCs

For murine MSCs, non-permeabilized bone marrow cells collected from mice were incubated with the antibodies against MSCs cell surface markers including anti-CD45-APC, anti-Ter119-APC, anti-CD31-APC, and anti-LepR-biotin. For cell surface CD36, LRP5 and LRP6 detection, anti-CD36 (Biolegend, 102606, 1:200), anti-LRP5 (Abcam, ab203201, 1:200) or anti-LRP6 [EPR2423(2)] (Abcam, ab134146, 1:200) were added individually to the non-permeabilized cells followed by FITC-conjugated secondary antibody. The frequency of CD36-, LRP5-, or LRP6-positive cells in the total LepR^+^CD45^–^CD31^–^Ter119^-^ cell population was calculated.

To detect cell surface CD36, LRP5 and LRP6 expression in human MSCs, non-permeabilized human MSCs purchased from Lonza were incubated with individual antibodies: anti-CD36 (Biolegend, 102606, 1:200), anti-LRP5 (Abcam, ab203201, 1:200) or anti-LRP6 [EPR2423(2)] (Abcam, ab134146, 1:200) followed by FITC- and PE-conjugated secondary antibody. The frequency of CD36-, LRP5-, or LRP6-positive cells in the total MSCs was calculated. Flow cytometry analyses were performed using a LSR II flow cytometer.

### In-vivo analysis of Dil-nLDL and Dil-oxLDL uptake by bone marrow MSCs

Mice were injected intravenously with 1, 1′-dioctadecyl-3,3,3′3′-tetrametryl-indocarbocyane perchlorate –labeled nLDL (DiI-nLDL) or oxLDL (DiI-oxLDL) 20 μg per mouse, 4 h before euthanasia. For flow cytometry analysis, bone marrow cells were flushed and red blood cells were removed as described above. CD45^+^ cells were removed by CD45 MicroBeads using MACS cell separation system (Miltenyi Biotec). Flow cytometric analyses were performed using a LSR II flow cytometer. The antibody used was anti-LepR-biotin (R&D Systems, AF497, 1:500) followed by a streptavidin-brilliant violet 421(Biolegend, 405225, 1:500). The uptake of DiI-nLDL and DiI-oxLDL by MSCs were analyzed by calculating the frequency of PE-DiI^+^ cells in the total CD45^-^LepR^+^ cell population.

### Flow cytometry analysis of in vitro Dil-nLDL and Dil-oxLDL uptake by human MSCs

Cells were cultured in lipoprotein-deficient medium (KALEN Biomedical LLC) for 12 h and then pre-chilled for 30 min at 4 °C. Cells were then incubated with DiI-nLDL or DiI-oxLDL at 20 μg·mL^-1^ in lipoprotein-deficient serum for 2 h at 4 °C, followed by another 30 min of incubation at 37 °C. DiI-nLDL or DiI-oxLDL uptake was analyzed by flow cytometry.

### Fluorescence microscopy

To visualize the binding and uptake of DiI-nLDL and DiI-oxLDL by MSCs, MSCs isolated from GFP transgenic mice were seeded on poly-D-lysine-coated cover glasses in a 6-well plate. Cells were cultured in lipoprotein-deficient medium for 12 h. For binding assays, cells were prechilled for 30 min at 4 °C. Cells were then incubated with DiI-nLDL or DiI-oxLDL at 20 μg·mL^-1^ in lipoprotein-deficient serum for 2 h at 4 °C. For the uptake assays, cells were pre-chilled for 30 min at 4 °C. Cells were then incubated with DiI-nLDL or DiI-oxLDL at 20 μg·mL^-1^ in lipoprotein-deficient serum for 2 h at 4 °C, followed by another 10 and 30 min of 37 °C incubation. Cells were fixed in 4% paraformaldehyde, permeabilized in 0.1% Triton X-100, mounted, and visualized under a confocal microscope (Zeiss LSM780).

Human MSCs were seeded on poly-D-lysine-coated cover glasses in a 6-well plate to visualize the subcellular co-localization of LRP6 with clathrin. Cells were treated with POVPC (Avanti Polar Lipids, Inc., 870606) at 5 µmol·L^-1^ for 0, 5, 10, and 30 min at 37 °C. Cells were fixed in 4% paraformaldehyde, permeabilized in 0.1% triton X-100, blocked in 1% BSA for 30 min, and incubated with anti-LRP6 [EPR2423(2)] (Abcam, ab134146, 1:600) and anti-Clathrin followed by (BD Biosciences, 610499, 1:200) FITC- and Texas Red–conjugated secondary antibodies, respectively. Specimens were then examined by confocal microscopy.

For time-lapse imaging of the co-endocytosis of LRP6 and oxLDL, HEK293T transfected with LRP6-eGFP^65^ were prechilled for 30 min at 4 °C. Cells were then incubated with DiI-nLDL or DiI-oxLDL at 20 μg·mL^-1^ in lipoprotein-deficient serum for 2 h at 4 °C. The culture dishes were then placed into the 37 °C microscope stage adapter, and confocal images were acquired using a spinning disk confocal imaging system based on a confocal microscope (Zeiss LSM780). Serial confocal images were recorded in the environmental chamber, which ensured a constant temperature, humidity and 5% CO_2_ atmosphere through the duration of imaging. The image integration time was 100 ms Intervals between image acquisition were 20–30 s.

### Fluorescence-based binding assays

The binding of fluorescence labeled lipids, DiI-oxLDL or PyrOVPC (Avanti Polar Lipids Inc.), to the MSC cell surface was detected as described previously.^66^ Briefly, 8 × 10^3^ human MSCs were seeded into 96-wells plate. After being cultured in lipoprotein-deficient medium for 12 h, the cells were pre-chilled for 30 mins at 4 ℃. Cells were then incubated with increasing concentration of DiI-oxLDL or PyrOVPC in the absence or presence of a 20-fold excess of unlabeled oxLDL or POVPC for 2 h at 4 °C. Cells were washed, fluorescence spectra detection was performed on a Synergy HTX Multi-Mode Microplate reader (Biotek). Excitation spectra were monitored at 530 nm for DiI-oxLDL and 360 nm for PyrOVPC, and emission spectra excited at 590 nm for DiI-oxLDL and 480 nm for PyrOVPC with all determinations at room temperature.

### PLO assays

PLO assays were performed as described previously.^45,46^ Briefly, 1 μl of nLDL or oxLDL at 20 µg·mL^-1^, or POVPC at 10 μg/ml was individually spot onto Hybond-C extra nitrocellulose membrane (Amersham) with three repeats per group. After drying and blocking, the membranes were incubated with either 1 μg·mL^-1^ protein lysates or purified GST-fusion proteins in blocking buffer. After washing, the membrane were incubated with individual primary antibodies followed by HRP-conjugated secondary antibody and ECL detection.

### Cell surface protein biotinylation

Cell surface protein biotinylation was performed as described previously.^67–69^ After being treated with different types of oxPLs, the cells were washed with PBS and incubated with 32 μL 10 mmol·L^-1^ Sulfo-NHS-SS-Biotin (Thermo Fisher) in 400 μL of reaction volume for 30 min at room temperature. Cells were washed twice with 100 mmol·L^-1^ glycine in PBS, lysed for 30 min on ice in 1% Triton-100 PBS and cell lysate centrifuged at 10 000 × *g* for 10 min at 4 °C. Equal amounts of cell lysates were incubated with 40 µL of streptavidin-agarose (Pierce Biotechnology) for 1 h at 4 °C. Beads were washed three times with ice-cold PBS and boiled in LDS sample buffer with 5% 2-mercaptoethanol. Samples were centrifuged for 1 min at 1 000×*g* and supernatants were subjected to western blot analysis with different antibodies.

### siRNA transfection, Western blot, and ELISA analysis of cAMP production

Cells were seeded at 60%–80% confluence at transfection. Within 24 h, individual siRNAs including control siRNA, LRP5 siRNA, and LRP6 siRNA (Ambion) were transfected using lipofectamin RNAiMAX reagent (Invitrogen) according to the manufacturer’s instruction. Western blot analysis of cell lysates was performed as described previously.^45,46^ The antibodies used were anti- LRP6 [EPR2423(2)] (Abcam, ab134146, 1:500), anti-β-actin (Cell Signaling, 3700, 1:1 000), anti-pCREB (Abcam, ab32096, 1:5 000), anti-CREB (Cell Signaling, 9197, 1:1 000), anti-pSmad2/3 (Cell Signaling, 8828, 1:1 000), anti-Smad2/3 (Cell Signaling, 3102, 1:1 000), anti-phospho-Smad1/5/8 (MilliporeSigma, AB3848, 1:1 000), anti-β-catenin (cell signaling, 9562, 1:1 000), anti-Na-K-ATPase (Abcam, ab7671, 1:1 000), and anti-Flag (Sigma-Aldrich, F3165, 1:2 000). All blots were developed by the enhanced chemiluminescence technique (Amersham). For intracellular cAMP measurement, an enzyme immunoassay kit (Cayman Chemical) was used, and the sample preparation and cAMP concentration detection were performed according to the manufacturer’s instruction.

### Quantitative real-time PCR

Total RNA for quantitative real-time PCR (qRT-PCR) was extracted from the cultured cells using Trizol reagent (Invitrogen) according to the manufacturer’s protocol. For qRT-PCR, cDNA was prepared with random primers using the SuperScript First-Strand Synthesis System (Invitrogen) and analyzed with SYBR GreenMaster Mix (QIAGEN) in the thermal cycler with two sets of primers specific for each targeted gene. Relative expression was calculated for each gene by the 2-CT method with GAPDH for normalization. Primers used for qRT-PCR were: RUNX2 (5′-CTGACCTCACAGATCCCAAGC-3′) and (5′-TGGTCTGATAGCTCGTCACAAG-3′); and Osterix (5′-TACCGGCCACGCTACTTTCTTTAT-3′) and (5′-GACCGCCAGCTCGTTTTCATCC-3′).

### Statistics

Data are presented as mean ± standard error of the mean. Unpaired, two-tailed Student’s *t*-tests were used for comparisons between two groups. For multiple comparisons, one-way analysis of variance (ANOVA) with Bonferroni post hoc test was used. All data were normally distributed and had similar variation between groups. For all experiments, *P* < 0.05 was considered significant. Statistical analysis was performed using SPSS, version 13.0 software (IBM Corp).

## Electronic supplementary material

Supplementary Figures
