# Supplementary material for: Oxidized phospholipids are ligands for LRP6
Source: Bone Res. 2018 Jul 18;6:22. doi: 10.1038/s41413-018-0023-x (PMC6050227; doi:10.1038/s41413-018-0023-x)
Supplement: Supplementary file 1 — Supplementary Figures [file 41413_2018_23_MOESM1_ESM.pdf]

Figure S1

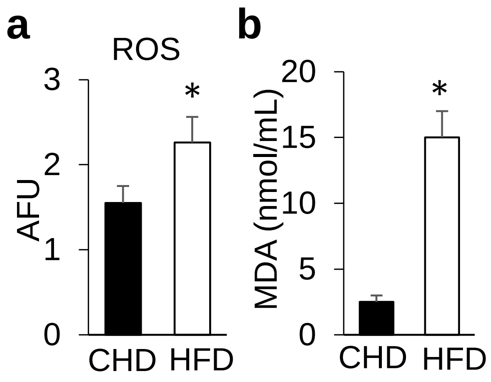

Figure S1. Lipid Oxidation is Increased in Mice Fed HFD.

Ten-week-old C57BL/6 mice fed Western high fat diets (HFD) or standard chow diets (CHD) for one month. Levels of ROS in MSCs (**a**) and MDA in serum (**b**) were detected. (n= 7, * $p<0.01$, vs. CHD group.)

Figure S2

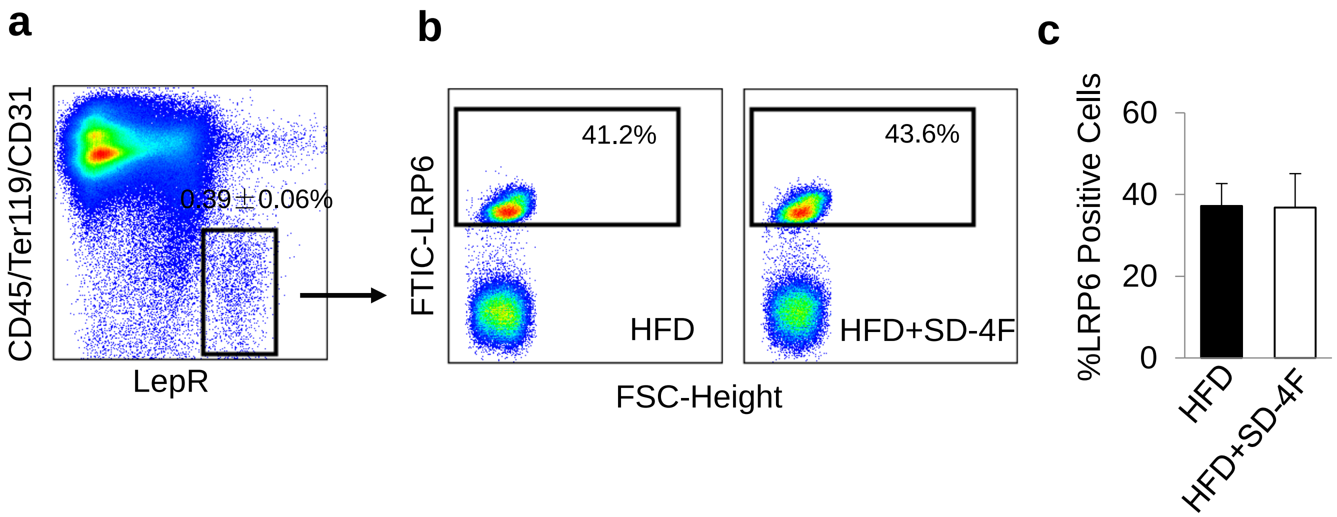

Figure S2. Control scrambled SD-4F peptide does not affect LRP6 cell surface expression in MSCs.

Ten-week-old C57BL/6 mice fed HFD alone or together with scrambled D-4F peptide (HFD+SD-4F) for two weeks. The peptides were administered orally in drinking water at 0.4 mg/ml, equivalent to 1.6 mg/d. Flow cytometry analysis of $LRP6^+$ cells in bone marrow $LepR^+CD45^-CD31^-Ter119^-$ MSCs (**a**). Representative images of the flow cytometry analysis (**b**) and the percentage of $LRP6^+$ cells (**c**) in MSCs. n=4, data are represented as mean±s.e.m.

Figure S3

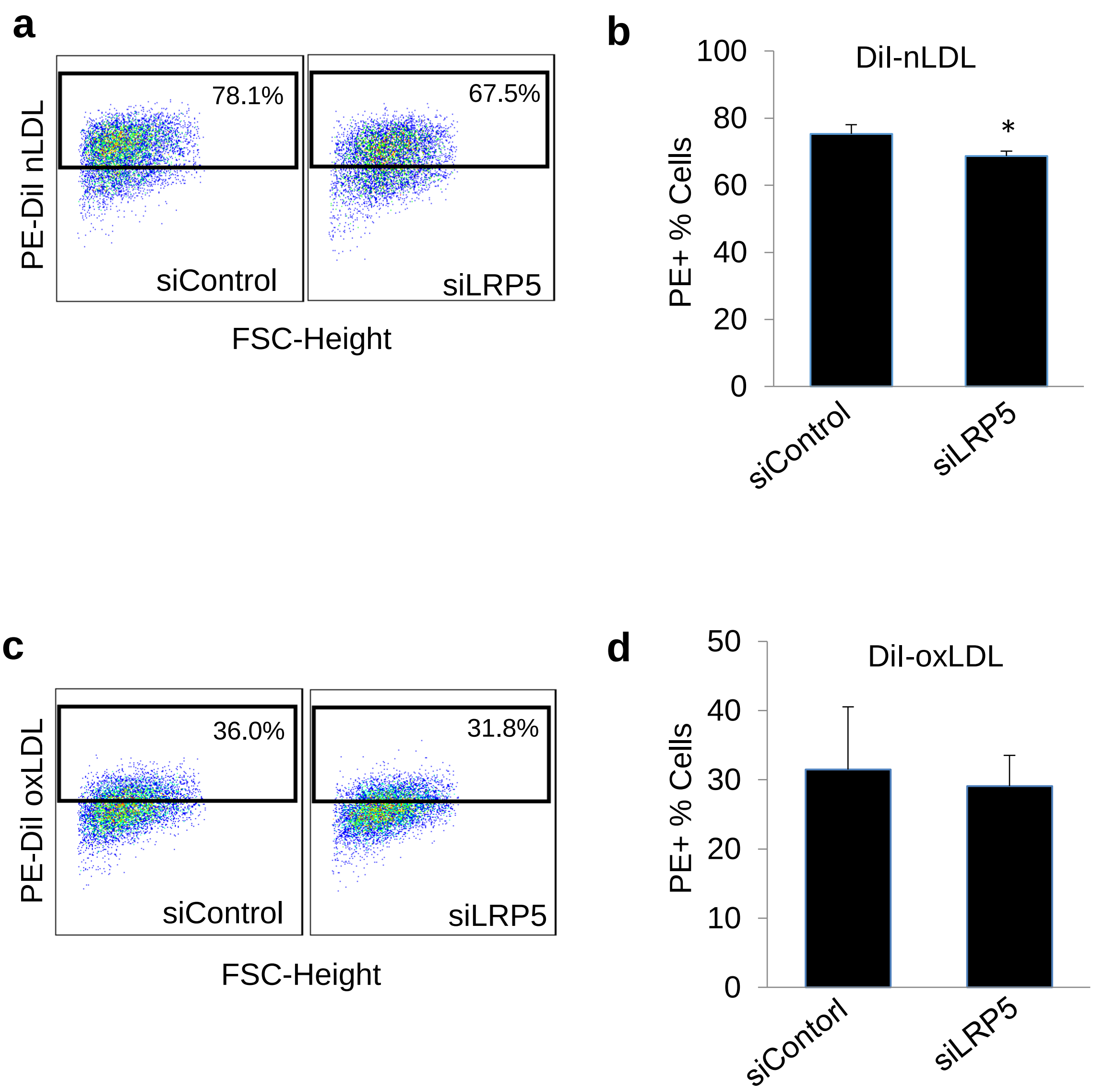

Figure S3. Uptake of DiI-nLDL by MSCs Were Reduced in MSCs with LRP5 Knockdown, But Were Not Affected the Uptake of DiI-oxLDL.

Representative images of the flow cytometry analysis (**a and c**) and the percentage of DiI-nLDL uptake (**b**) and DiI-oxLDL uptake (**d**) in human MSCs transfected with either irrelevant control siRNA or LRP5 siRNA. (n=6, data are represented as mean$\pm$s.e.m. *p<0.05, vs. Control siRNA group, as determined by Student's *t*-tests)

Figure S4

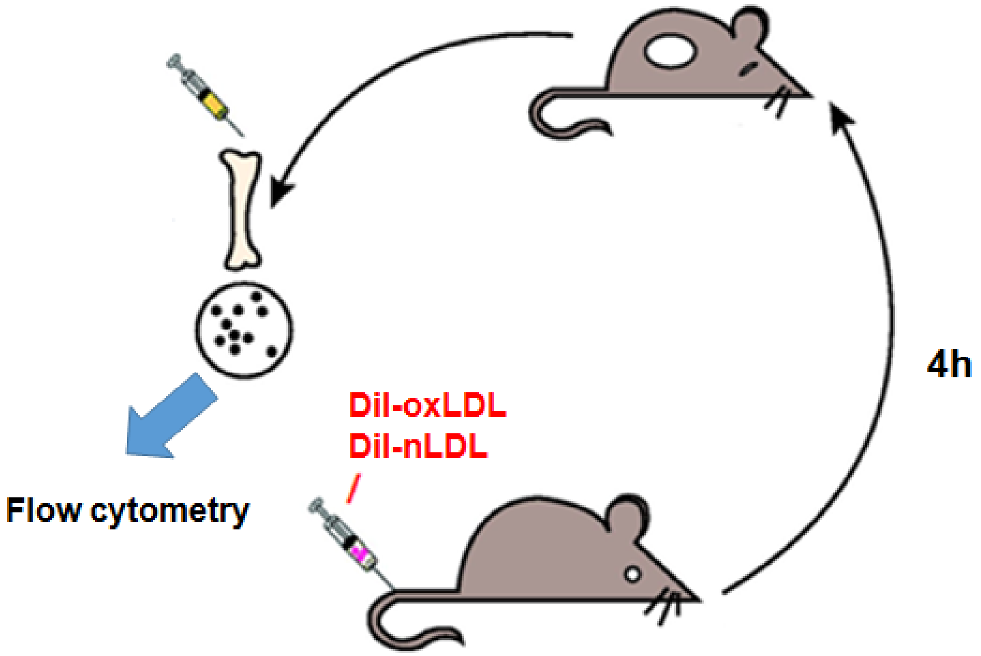

Figure S4. Schematic of Experimental Protocol Testing nLDL and oxLDL Uptake by Bone Marrow MSCs in Mice.

Mice were injected intravenously with DiI-nLDL or DiI-oxLDL 20 μg per mouse. After 4 hour, bone marrow MSCs were isolated and the uptake of DiI-nLDL and DiI-oxLDL by MSCs were analyzed by flow cytometry. Please also see details in the Methods section.
